# The Weathering Microbiome of an Outcropping Granodiorite

**DOI:** 10.3389/fmicb.2020.601907

**Published:** 2020-12-14

**Authors:** Stephanie A. Napieralski, Eric E. Roden

**Affiliations:** Department of Geoscience, University of Wisconsin-Madison, Madison, WI, United States

**Keywords:** iron oxidation, weathering, chemolithotrophy, metagenomics, extracellular electron transfer (EET)

## Abstract

Microorganisms have long been recognized for their capacity to catalyze the weathering of silicate minerals. While the vast majority of studies on microbially mediated silicate weathering focus on organotrophic metabolism linked to nutrient acquisition, it has been recently demonstrated that chemolithotrophic ferrous iron [Fe(II)] oxidizing bacteria (FeOB) are capable of coupling the oxidation of silicate mineral Fe(II) to metabolic energy generation and cellular growth. In natural systems, complex microbial consortia with diverse metabolic capabilities can exist and interact to influence the biogeochemical cycling of essential elements, including iron. Here we combine microbiological and metagenomic analyses to investigate the potential interactions among metabolically diverse microorganisms in the near surface weathering of an outcrop of the Rio Blanco Quartz Diorite (DIO) in the Luquillo Mountains of Puerto Rico. Laboratory based incubations utilizing ground DIO as metabolic energy source for chemolithotrophic FeOB confirmed the ability of FeOB to grow via the oxidation of silicate-bound Fe(II). Dramatically accelerated rates of Fe(II)-oxidation were associated with an enrichment in microorganisms with the genetic capacity for iron oxidizing extracellular electron transfer (EET) pathways. Microbially oxidized DIO displayed an enhanced susceptibility to the weathering activity of organotrophic microorganisms compared to unoxidized mineral suspensions. Our results suggest that chemolithotrophic and organotrophic microorganisms are likely to coexist and contribute synergistically to the overall weathering of the *in situ* bedrock outcrop.

## Introduction

The weathering of Earth’s continental crust involves a complex set of physical, geochemical, and biological reactions. As microorganisms are ubiquitous in soils and sedimentary environments, often preferentially associated with mineral surfaces (Hazen et al., 1991) they have vast potential to enhance mineral weathering thus impacting the cycling of bioessential elements between the lithosphere and the biosphere. Microbiological impacts on weathering processes, from the aid in physical disaggregation by mechanical forcing to enhancement of chemical dissolution have long been recognized and are extensively described (Banfield et al., 1999; Uroz et al., 2009). Well established mechanisms of microbially enhanced mineral dissolution revolve around organotrophic metabolisms, where microorganisms solubilize mineral components to meet their nutritional needs for conveyance of a growth benefit (Bennett et al., 2001) and provide valuable ecosystem services for higher biota (Calvaruso et al., 2006; Lambers et al., 2009). Acidolysis and chelation by organic acids (Drever and Stillings, 1997; Uroz et al., 2009) as well as siderophores (Kalinowski et al., 2000; Liermann et al., 2000; Buss et al., 2007) have been extensively invoked when relating microbial activity to mineral weathering.

Redox active elements, such as Fe, are often present in igneous rocks. If Fe constitutes a considerable component of the mineral structure, redox reactions can occur prior to bulk dissolution if the kinetics of electron transfer are faster than structural disintegration (White, 1990). Oxidation of structural Fe(II) is in fact often rate limiting in terms of Fe(II) mineral weathering (Hering and Stumm, 1990). Fe(II) silicate minerals in fresh rock, in disequilibrium with Earth’s oxidizing surficial environment, represent a vast supply of electrons to potentially fuel microbial metabolism and growth, including by chemolithotrophic Fe(II)-oxidizing bacteria (FeOB). FeOB are known to occupy distinct environmental niches where opposing gradients of ferrous iron [Fe(II)] and oxidants (e.g., O_2_) intersect, such as aquatic sediments, freshwater iron seeps, and hydrothermal vents (Sobolev and Roden, 2002; Roden et al., 2004; Emerson et al., 2010). By analogy, the interface between reduced igneous rock and the oxidizing atmosphere represents such a gradient, albeit a very sharp, solid phase one, that theoretically can provide energy to fuel biomass production (Jakosky and Shock, 1998; Buss et al., 2005; Shock, 2009). While studies have suggested that FeOB are capable of direct utilization of the structural Fe(II) in silicate minerals (Popa et al., 2012; Roden, 2012; Shelobolina et al., 2012b) and glasses (Bailey et al., 2009; Henri et al., 2015), only recently has the ability of FeOB to utilize crystalline silicate-bound Fe(II) in rocks for metabolic energy generation and subsequent growth been demonstrated (Napieralski et al., 2019). By combining microbiological and metagenomic based approaches, Napieralski et al. (2019) demonstrated that FeOB can dramatically accelerate the oxidation of silicate mineral bound Fe(II) via extracellular electron transfer (EET) coupled to cellular growth, and that the subsequent oxidative weathering of the minerals biotite and hornblende within granitic rocks resulted in subtle changes to the surface structure that rendered the mineral more susceptible to proton promoted dissolution via dilute acid. While not explicitly demonstrated, the redox driven mineralogical transformations associated with FeOB activity should then also affect the efficiency at which organotrophic microorganisms are able to solubilize cations for nutritional purposes.

**FIGURE 1:**
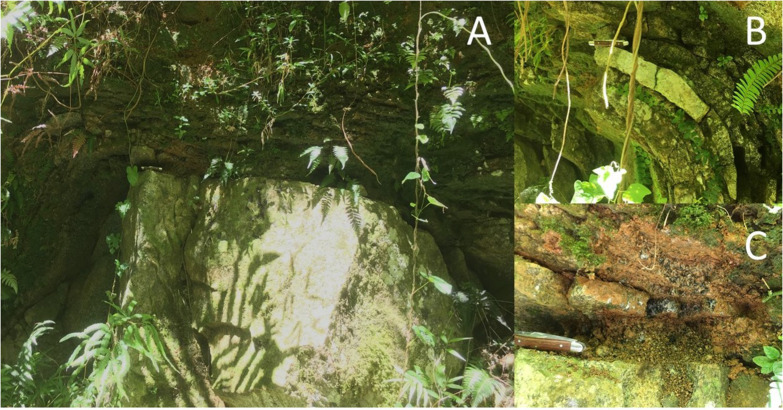
Outcrop scale photo of a weathering corestone of Rio Blanco Quartz Diorite exposed by road cutting **(A)**. The corestone exhibits spheroidal weathering, with partially altered “rindlets” surrounding the unaltered rock **(B)**. Material for this study was collected near the rindlet-corestone interface **(C)**. Pocket knife for scale (ca. 9 cm).

It is important to consider the entire suite of biogeochemical reactions that may be mediated by complex microbial communities in natural systems. Our previous work specifically investigated the role of chemolithotrophic FeOB in the subsurface (ca. 8 m) weathering of the Rio Blanco Quartz Diorite (Napieralski et al., 2019), well below the rooting zone where organic carbon content is minimal. In contrast, the near surface, where inputs from plant derived organic matter are of substantial concern (Banfield et al., 1999), a more complex interplay between organotrophically and lithotrophically mediated processes may be involved in the weathering of Fe(II)-silicates. In order to address this potential interplay, samples were collected for enrichment culturing and metagenomic analysis from near-surface outcrop of the rapidly weathering Rio Blanco Quartz Diorite of the Luquillo Mountains, Puerto Rico (Buss et al., 2008; Figure 1). After establishment, Fe(II)-silicate oxidizing enrichment cultures were amended with additional carbon to assess the degree to which prior lithotrophic activity might enhance the cation solubilizing ability of organotrophic microorganisms. Our results indicate that chemolithotrophic and organotrophic microorganisms can coexist and work synergistically to enhance the weathering of near surface silicate rock.

## Materials and Methods

### Field Sampling

In June of 2017, samples were collected from a road cut exposure of the Rio Blanco Quartz Diorite (abbreviated hereafter as “DIO”) previously described by Buss et al. (2008) (See Figure 1). Rindlets nearest the weathering corestone were carefully excavated using a rock hammer and spatula previously sterilized by ethanol soaking and flame and placed into sterile Whirlpack bags. Samples were then placed in coolers and shipped overnight to The University of Wisconsin-Madison where portions were promptly placed at −80°C and 4°C for DNA extraction and enrichment culturing, respectively.

### Chemolithotrophic Enrichment Culturing

Chemolithotrophic Fe(II)-oxidizing cultures were established as previously described with DIO and Fe(II)-free quartz sand (QTZ) (Napieralski et al., 2019). Briefly, 5.0 g of ground and sieved (<45 μM) DIO or quartz sand (Acros Chemicals 140–381 μm) were added to 50 mL of Luquillo artificial ground water (L-AGW), autoclaved anoxically, and aerated with sterile air. 5 v/v% CO_2_ was added to the headspace and the appropriate bottles were inoculated with ca. 1.0 g of material from the DIO outcrop that had been aseptically fragmented. No mineral amendment controls were also prepared by adding ca. 1.0 g of inocula to 50 mL of L-AGW without additional minerals (NoMin). Samples were taken immediately following inoculation and at 41, 82, 105, 130, 182, 252, 334, and 615 days for analyses as described below.

### Organotrophic Incubations

**FIGURE 2:**
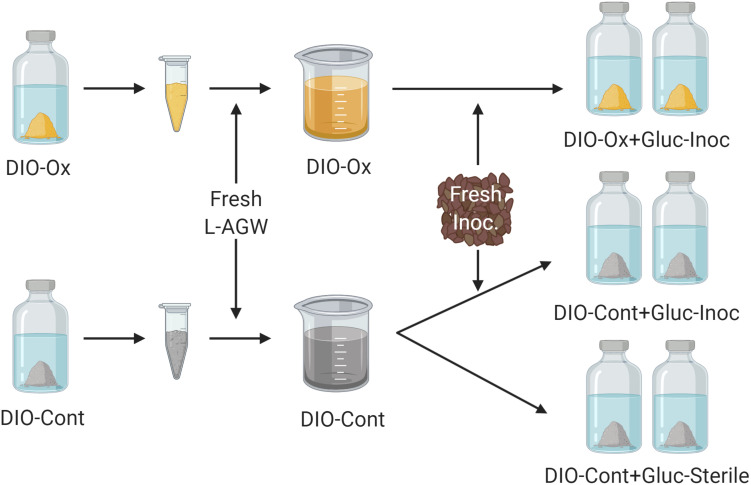
Conceptual cartoon of the generation of organotrophic incubations. The solids were isolated from the initial lithotrophic incubations for both live inoculated (DIO-Ox) and uninoculated control experiments (DIO-Cont) and resuspended in fresh Luquillo artificial river water (L-AGW). The resultant slurries were divided into duplicate reactors, and either reinoculated (Inoc) or left as a sterile control and stoppered. 1.0 mM filter sterilized glucose (Gluc) was added to each reactor via needle and syringe.

After 615 days of initial chemolithotrophic incubation, 5.0 mL of DIO from one replicate (to ensure consistency of substrate) of both live/inoculated and abiotic control reactors was removed and the solids separated via centrifugation. The aqueous phase was discarded, and the solids were resuspended in 40 mL of fresh L-AGW. The resultant slurries (temporarily denoted as DIO-Ox and DIO-Cont) were each transferred in equal amounts (20 mL) to two sterile bottles. The bottles were reseeded with ca. 0.1 g of DIO outcrop material and 1.0 mM of filter sterilized glucose was added; these reactors are referred to as DIO-Ox + Glu-Inoc and DIO-Cont + Glu-Inoc. For abiotic controls, 5.0 mL of the second replicate of the abiotic control from the chemolithotrophic incubations was similarly prepared in fresh L-AGW with glucose but was left uninoculated; these reactors are referred to as DIO-Cont + Gluc-Sterile (see Figure 2). Samples were taken at T_0_, and after 1, 3, 14, 28, and 60 days of incubation for the analyses described below.

### Analytical Methods

#### ATP

ATP content of enrichment cultures was determined via luminescence using BacTiter-Glo^TM^ (Promega, Madison, WI, United States) as previously described (Napieralski et al., 2019).

#### Solid-Phase Fe(II)

The ratio of Fe(II) to total Fe released by 0.5 M HCl extraction was determined on the solid phase of 1.0 mL of total enrichment culture subsamples. The solids were separated via centrifugation and extracted for 24 h in 5 mL of 0.5 M HCl on an orbital shaker. Fe(II) of each extract was determined by the standard Ferrozine assay (Stookey, 1970) and the measurement was repeated after the addition of hydroxylamine-HCl for determination of Fe(total).

#### Cations

The aqueous phase from duplicate reactors was pooled, diluted 1:5 in 5 mM HNO_3_ and filtered through a 0.22 μm filter. Major cation concentrations (Ca, K, Mg, and Na) were determined using inductively coupled plasma optical emission spectroscopy (ICP-OES).

#### Particulate Organic Matter

The POC content of triplicate samples of the weathered material at the rindlet-corestone interface (see Figure 1C) used to establish initial enrichment cultures was determined via high temperature combustion utilizing a Flash EA 1112 Flash Combustion Analyzer.

### DNA Extraction, Metagenomic Sequencing, and Assembly

DNA was extracted from duplicate outcrop (Gb-OC) and 182 days enrichment culture material (DIO-Inoc, QTZ-Inoc, and NoMin-Inoc) utilizing previously described (Napieralski et al., 2019) adaptations to the SDS-based DNA extraction method of Zhou et al. (1996). DNA was submitted to the University of Wisconsin-Madison Biotechnology Sequencing Center for shotgun metagenomic library preparation and 2 × 250 sequencing on the Illumina HiSeq 2500 rapid platform. Raw reads were quality filtered using the default parameters Trim-Galore. Concatenated reads from all samples were assembled using IDBA-UD (Peng et al., 2012) utilizing the high-performance computing cluster at the Center for High Throughput Computing at University of Wisconsin-Madison.

### Metagenomic Analysis

Metagenome assembled genomes (MAGs) were obtained from the metagenomic co-assembly using the Bin Refinement module of metaWRAP (Uritskiy et al., 2018) with initial bin sets generated using MetaBat2 (Kang et al., 2019) and Concoct (Alneberg et al., 2014). Quality, completion and initial taxonomy of refined MAGs were assessed using CheckM (Parks et al., 2015). Final consensus taxonomy of MAGs was determined using the Classify Bins module of metaWRAP and extraction of essential single-copy genes as described by He et al. (2016) for each MAG. The relative abundance of each MAG (genomes per million reads) across all samples in the co-assembly was determined using the Bin Quantification module of metaWRAP. Open reading frames (ORFs) were predicted and annotated using Prokka (Seemann, 2014). Subcellular location of putative proteins was predicted using Cello (Yu et al., 2006). Putative extracellular electron transfer (EET) pathways for iron oxidation were identified as previously described (He et al., 2017) using BLASTP and HMMsearch for homologs to the Cyc2-type system of *Acidithiobacillus ferrooxidans* (Castelle et al., 2008) and MtoAB of *Sideroxydans lithotrophicus* ES-1 (Liu et al., 2012). Putative siderophore biosynthesis pathways were identified using HMMsearch for the PFAMs associated with non-ribosomal peptide synthesis (NRPS) condensation and adenylation domains as well as the conserved IucAC domains of NPRS-independent synthesis (NIS) (Hopkinson and Barbeau, 2012). Selected MAGs were investigated for the presence of carbohydrate active enzymes using the dbCAN webserver (Yin et al., 2012).

## Results

### Initial Chemolithotrophic Enrichment

**FIGURE 3:**
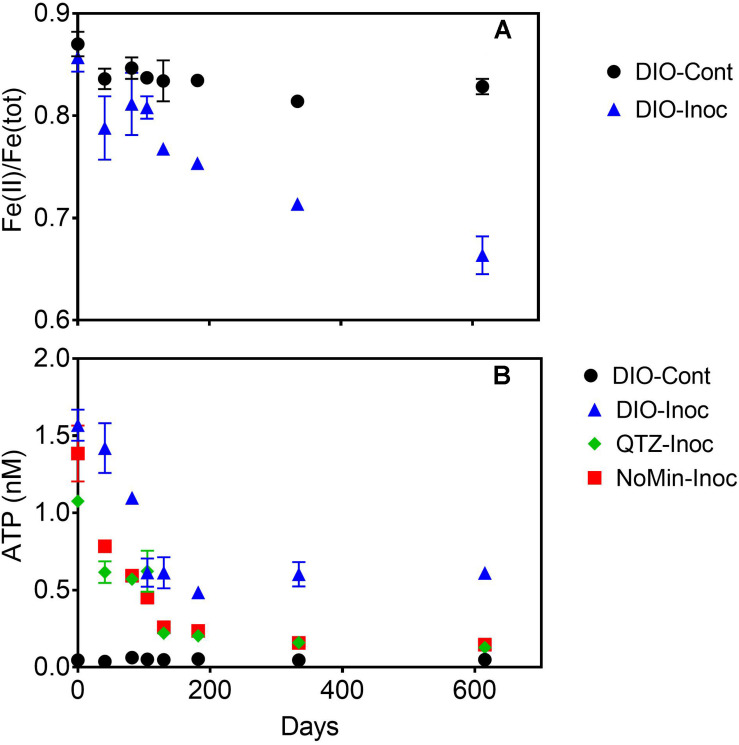
Fe(II)/Fe(tot) of dilute HCl extractable Fe in abiotic uninoculated (DIO-Cont) and live inoculated (DIO-Inoc) diorite enrichment cultures **(A)**. ATP content (nM) of control DIO and inoculated DIO, quartz (QTZ), and inocula only (NoMin) cultures **(B)**. Data point and error bars denote the mean and range of duplicate reactors.

Over the course of the 615-days chemolithotrophic incubation, Fe(II)/Fe(total) declined from an average of 0.857 to 0.664 in the live DIO reactors; no systematic change in Fe(II)/Fe(total) was observed in sterile, abiotic controls (Figure 3A). The ATP content of all inoculated reactors decline precipitously from initial values of ca. 1.3 nM over the first 130 days of incubation (Figure 3B). In DIO cultures, ATP content stabilized at an average of ca. 0.6 nM for the duration of the experiment, whereas ATP continued to decline to ca. 0.13 and 0.15 nM in QTZ and NoMin (inoculum only) cultures, respectively. No net change in background ATP (abiotic control) was observed.

**FIGURE 4:**
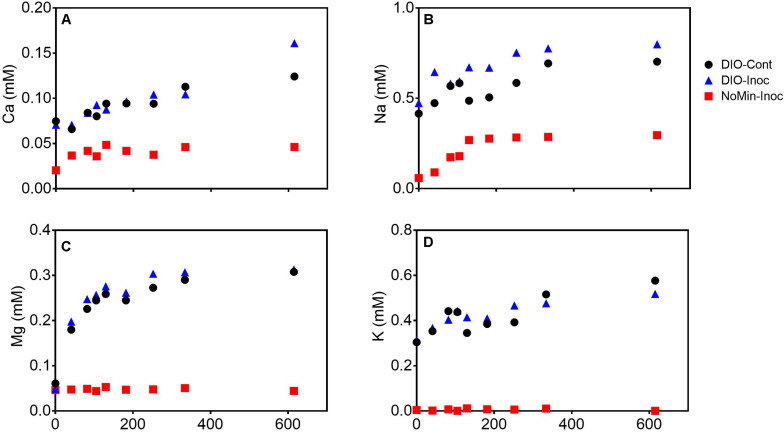
Ca **(A)**, Na **(B)**, Mg **(C)**, and K **(D)** concentrations (mM) in pooled duplicate control (DIO-Cont), live inoculated (DIO-Inoc), and inocula only (NoMin-Inoc) reactors.

The release of major rock forming cations (Ca, Mg, K, and Na) was detected in all DIO amended reactors (Figure 4). Both Ca and Na concentrations showed a modest increase in net release in inoculated reactors relative to sterile abiotic controls (Figures 4A,B). Final Ca concentrations reached 0.124 and 0.160 mM in control and live inoculated reactors, respectively, corresponding to increases in concentration of 50 and 91 μM Ca over the course of the experiment. Final Na concentrations increased to 0.288 and 0.326 mM in the control and live reactors, respectively. Thus, 41 and 37 μM more Ca and Na, respectively, was released in the presence of a live inoculum relative to sterile controls. Ca and Na release was also detected in the inoculum only NoMin reactors, indicating release from DIO in the inoculum. No difference in Mg concentration between control and live reactors was observed, nor was there any release of Mg in NoMin reactors (Figure 4C). K release was also not detected in NoMin reactors, and in contrast to Ca and Na, overall release of K was higher in abiotic reactors relative to live inoculated (Figure 4D), with 0.06 mM more K released in abiotic reactors over the course of the experiment.

### Organotrophic Incubation

**FIGURE 5:**
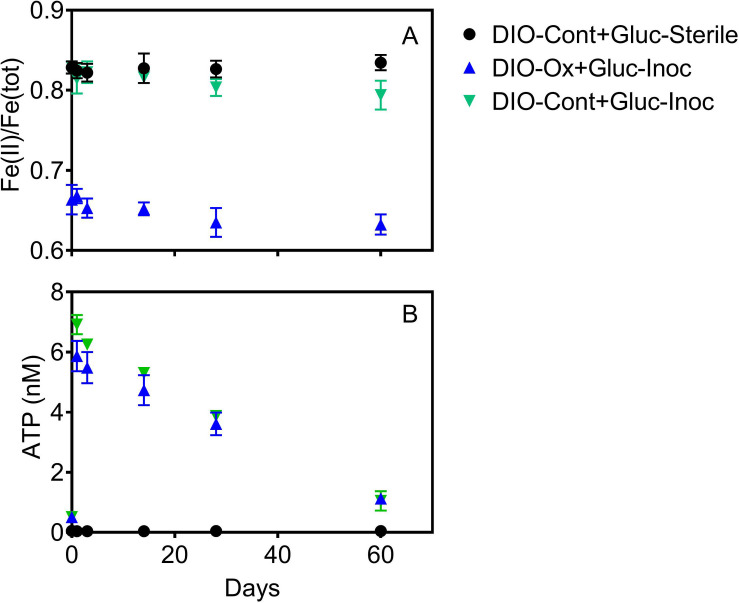
Fe(II)/Fe(tot) **(A)** and ATP content **(B)** after supplementation with 1.0 mM glucose in live cultures containing microbially oxidized DIO from the previous chemolithotrophic enrichment culture (DIO-Ox-Gluc-Inoc), live cultures containing unoxidized DIO from previous abiotic controls (DIO-Cont-Gluc-Inoc), or sterile controls containing unoxidized DIO from previous abiotic controls (DIO-Cont + Gluc-Sterile).

Addition of glucose to stimulate organotrophic metabolism after imposed chemolithotrophic conditions resulted in immediate cell growth, with ATP increasing to 6–7 nM in live reactors after 1 day (Figure 5B). After initial growth, ATP content of the reactors declined over the remaining 60 days to an average final concentration of ca. 1.1 nM. Small changes were observed in Fe(II)/Fe(tot) for both glucose amended reactor sets, declining from an average of 0.664 to 0.633 in live cultures containing microbially oxidized DIO from the previous chemolithotrophic enrichment culture (DIO-Ox + Gluc-Inoc), and from an average of 0.829 to 0.794 in live cultures containing unoxidized DIO from previous abiotic controls (DIO-Cont + Gluc-Inoc) (Figure 5A). No oxidation was observed in the glucose-amended abiotic control (DIO-Cont + Gluc-Sterile) containing DIO from previous abiotic controls.

**FIGURE 6:**
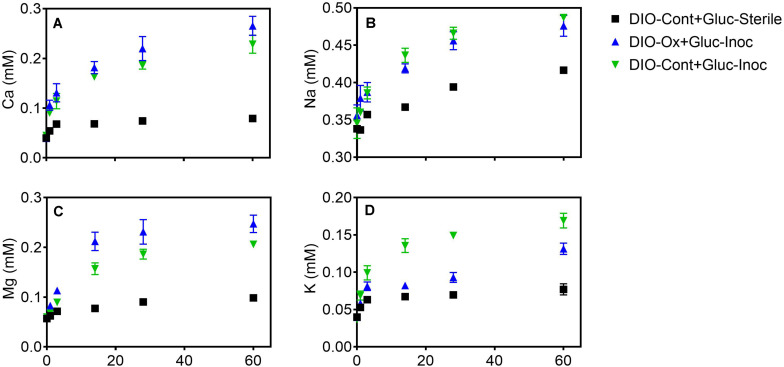
Ca **(A)**, Na **(B)**, Mg **(C)**, and K **(D)** concentrations after supplementation with 1.0 mM glucose in live cultures containing microbially oxidized DIO from the previous chemolithotrophic enrichment culture (DIO-Ox-Gluc-Inoc), live cultures containing unoxidized DIO from previous abiotic controls (DIO-Cont-Gluc-Inoc), or sterile controls containing unoxidized DIO from previous abiotic controls (DIO-Cont + Gluc-Sterile).

The release of all cations was stimulated in live cultures containing both previously oxidized and unoxidized DIO relative to unoxidized abiotic controls (Figure 6). Average final concentrations and total release of Ca and Mg were slightly higher (ca. 0.04 mM) in live cultures containing previously oxidized DIO compared to unoxidized (Figures 6A,C). The opposite trend was observed in K release, with final K concentrations being an average of 0.040 mM higher in DIO-Cont + Gluc-Inoc reactors (Figure 6D). Final average Na concentrations overlapped within the error of the replicates, though an average of 0.022 mM more Na was released in DIO-Cont + Gluc-Inoc vs. DIO-Ox + Gluc-Inoc reactors (Figure 6B).

### Metagenomic Analysis

**FIGURE 7:**
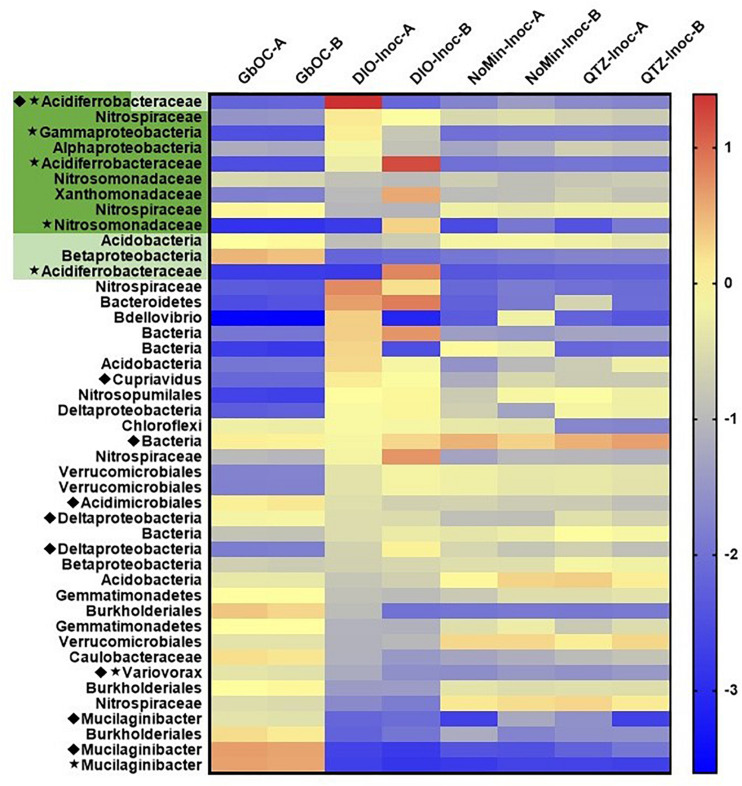
Heat map of the abundance (log genomes per million reads) of selected MAGs (the 10 most abundant MAGs in each sample and all MAGs with EET and siderophore biosynthesis pathways) across all samples in the metagenomic co-assembly including duplicate *in situ* outcrop (GbOC) and duplicate inocula only (NoMin-Inoc), quartz (QTZ-Inoc) and diorite (DIO-Inoc) enrichment cultures. Putative FeOB are highlighted, with the presence of Cyc2 homologs in dark green and MtoAB in light green. The presence of RuBisCO is indicated by a star and siderophore biosynthesis by a diamond.

A total of 84 MAGs (>70% complete, <10% redundant) were obtained from the metagenomic co-assembly. Of these MAGs, 12 contained homologs to EET pathways putatively involved in Fe(II) oxidation, with nine MAGs containing Cyc2 homologs, three containing MtoAB, and one containing both. Of these putative FeOB MAGs, five also contained RuBisCO (Figure 7). In reactors amended with DIO, putative chemolithotrophic FeOB were enriched relative to the *in situ* samples and diorite free (NoMin and QTZ) reactors (Figure 7). The most abundant MAG in each replicate of the diorite oxidizing cultures was a putative chemolithoautotrophic γ-proteobacteria, most closely affiliated with the family *Acidiferrobacteraceae*. However, each replicate was dominated by a different *Acidiferrobacteracea*e MAG. While the most abundant MAG in DIO-OC-A contained homologs to both Cyc2 and MtoAB, the dominant MAG in DIO-OC-B contained only Cyc2. Putative siderophore biosynthesis pathways were identified in 9 MAGs (Figure 7). Compared to the diorite oxidizing enrichment cultures, MAGs containing putative siderophore biosynthesis pathways are overall more abundant *in situ.*

The two most abundant MAGs in both *in situ* libraries is a Sphingobacteriaceae, putatively of the genus *Mucilaginibacter* (Figure 7). In addition to containing a putative NRPS-type siderophore biosynthesis pathway, the *Mucilaginibacter* MAGs are enriched in carbohydrate active enzymes, particularly glycosyltransferases and glycoside hydrolases. Also abundant are β-proteobacteria, with four of the top 10 most abundant MAGs in both *in situ* metagenomes belonging to this class, three of which to the order *Burkholderiales*. In addition, two of the top 10 MAGs contain putative EET pathways but did not contain RuBisCO or other carbon fixation pathway, an MtoAB in a β-proteobacteria and Cyc2 in a *Nitrospiraceae*.

## Discussion

### Initial Chemolithotrophic Enrichments

The results of this study confirm the previously reported (Napieralski et al., 2019) ability of chemolithotrophic microorganisms from the Rio Blanco quartz diorite weathering environment to catalyze oxidation of Fe(II)-silicate phases in fresh rock (Figures 3A, 5A). In contrast to our previous findings, biologically enhanced release of Ca and Na was observed during initial chemolithotrophic incubation under all experimental conditions, including during incubation of endogenous DIO in the inoculum. Our 2019 study on the potential role of FeOB in DIO weathering utilized inocula from the subsurface rindlet-saprolite interface, well below the rooting zone where organic carbon content is very low (Buss et al., 2005). As such, transfer of labile organics and actively metabolizing organotrophic cells upon inoculation of enrichment cultures was likely to have been limited. The present study is thus fundamentally different in that the initial inoculum was obtained from a surficially exposed outcrop with visible roots and vegetation (see Figure 1), and organic matter was transferred into the enrichment cultures upon inoculation. The addition of 1.0 g of natural inoculum with an average POC content of 0.269 ± 0.012 wt% to the 50 mL reactors would result in an initial average organic carbon content of 4.48 ± 0.18 mM, representing a maximum possible amount of carbon, though the bioavailable fraction may be less. While it is well established that microorganisms can enhance the release of major rock forming cations from granitic material in the presence of glucose amendments (Wu et al., 2008; Frey et al., 2010), similar results have also been noted in a study of granitic gneisses and diorites incubated with only natural sediment and glacial waters without carbon or other nutrient amendment (Montross et al., 2013). These results indicate that even small amounts of naturally derived, potentially more complex forms of organic carbon are capable of supporting microbially enhanced mineral dissolution.

Due to the input of carbon and active organotrophic biomass in the inoculum, we are presently unable to directly link oxidation of the DIO to FeOB growth. ATP concentrations declined over time in all inoculated reactors (Figure 3), which suggests that microbial growth coupled to Fe(II) oxidation was insufficient to compensate for the decline in organotrophic biomass initially present in the inoculum. These results are consistent with a recent study utilizing microbially colonized shale for the establishment of chemolithotrophic pyrite-oxidizing enrichment cultures (Napieralski, 2020). Nevertheless, the rate and extent of the decline in ATP concentration was lower in reactors amended with DIO compared to those without added DIO (QTZ and NoMin; see Figure 3), suggestive of ATP generation linked to chemolithotrophic metabolism. The final “steady-state” ATP concentration of ca. 0.6 nM observed here is comparable to that reported by Napieralski et al. (2019) in the previous study on the chemolithotrophic oxidation of the DIO, where growth yield calculations were consistent with previously reported for yields for FeOB in opposing gradient media (Sobolev and Roden, 2004). Thus, the accelerated oxidation of mineral-associated Fe(II) (Figure 2) and presence and enrichment of multiple MAGs containing Fe(II)-oxidizing EET systems (Figure 7) is consistent with the growth of chemolithotrophic FeOB in the present study.

### Putative Chemolithotrophic Pathways

In both replicates of the diorite oxidizing enrichment cultures, the most abundant MAG belonged to the family *Acidiferrobacteraceae* (Figure 7) and contained a Cyc2 homolog as ribulose 1,5-bisphosphate carboxylase/oxygenase (RuBisCO), indicating their ability to grow chemolithoautotrophically. The family *Acidiferrobacteraceae* is currently described as harboring acidiphilic Fe and S oxidizers (Issotta et al., 2018), as well as neutrophilic S oxidizers (Kojima et al., 2015, 2016). Members of the genus *Acidiferrobacter* encompassing the acidophilic FeOB have been shown to contain homologs to Cyc2 as well as rusticyanin (Issotta et al., 2018), an acid-stable copper protein utilized by *Acidithiobacillus ferrooxidans* for electron transfer during Cyc2 dependent Fe(II) oxidation (Castelle et al., 2008), but not present in neutrophilic FeOB genomes (Barco et al., 2015; He et al., 2017; McAllister et al., 2020). Rusticyanin homologs were not identified in any of the *Acidiferrobacteraceae* MAGs obtained in this study. While there are currently no described neutrophilic FeOB within the *Acidiferrobacteraceae*, Meier et al. (2019) identified 16S rRNA gene sequences related to *Acidiferrobacteraceae* as being potentially involved in soil formation at a site selected to be free of influence of sulfides and where the pH was circumneutral. As our knowledge of the diversity of metabolic capacity of FeOB is rapidly expanding with the increase of environmental metagenomic data, the possibility remains that there are previously unrecognized neutrophilic FeOB within the family *Acidiferrobacteraceae.*

The taxonomy of the putative FeOB MAGs obtained in this study largely varied from the MAGs obtained from the subsurface weathering cultures, which included β-proteobacteria of the genus *Cupriavidus* and order Burkholderiales (Napieralski et al., 2019). A notable exception is the presence of a highly abundant *Xanthomonadaceae* MAG closely related to the non-autotrophic soil bacterium *Dyella japonica* A8 (Chen and Chan, 2012) which we previously identified as containing a homolog to Cyc2. Interestingly, Uroz et al. (2009) noted the ability of a *Dyella* sp. to solubilize biotite and other *Dyella* sp. have been isolated or identified from weathering environments (Uroz et al., 2011; Zhao et al., 2013), though no genomes are available to assess whether they contained Cyc2 homologs. In addition to the *Xanthomonadaceae* MAG, four additional MAGs identified in this study contained putative EET pathways but lacked RuBisCO. While alternative carbon fixation pathways exist, they tend to be phylogenetically restricted, with the vast majority of α, β and γ-proteobacteria utilizing the Calvin Cycle (Hugler and Sievert, 2011). Two of the MAGs containing EET but not RuBisCO belong to organisms of the phylum *Nitrospirae*, which are known to use the reductive tricarboxylic acid cycle (rTCA) for carbon assimilation. Although the rTCA cycle shares many of the same genes as the TCA cycle, the unique enzyme 2-oxoglutarate synthase can be used to distinguish the two pathways (Hugler and Sievert, 2011). As this gene was not present in either *Nitrospirae* MAG, it seems likely that they cannot grow autotrophically. The differences in the taxonomy of the putative chemolithotrophic FeOB identified in this study compared to the study on subsurface weathering of the DIO suggests that the ability to utilize mineral-bound Fe(II) for metabolic energy generation may not be a unique feature of any given FeOB. Rather, it may be a trait shared among FeOB, an idea supported by previous studies where phylogenetically diverse bacteria, putatively FeOB, were isolated on Fe(II)-phyllosilicate minerals (Shelobolina et al., 2012a; Benzine et al., 2013).

### Effect of Prior Oxidation on Mineral Weatherability

In line with previous observations on organotrophically driven mineral weathering (Bennett et al., 2001; Wu et al., 2008), the addition of organic carbon stimulated the release of major cations relative to sterile controls (Figure 6). Cellular uptake has been shown to be a potential sink for cations during the microbial weathering of basaltic rock (Stranghoener et al., 2018). However, the stimulated cation release observed here was probably not influenced significantly by cellular uptake, as a maximum of a few μM of Ca, K, Mg, and Na would be required for nutritional purposes given the relatively low cell densities in our cultures, [ca. 10^6^–10^7^ cells mL^–1^ for the chemolithotrophic and organotrophic enrichments, respectively, based on measured ATP concentrations of the cultures and typical cellular ATP contents (Balkwill et al., 1988)] and the typical elemental composition of bacterial cells (Heldal et al., 1985). Likewise, although bacterial cells sorb major cations and silica, the affinity of cells for these ions is much lower than for transitions metals and metalloids (Urrutia, 1997), and this process is not expected to have significantly altered aqueous speciation in our experiments. In any case, due to the possibility of cellular uptake and sorption to cell surfaces, the enhanced release of cations observed in our experiments represents a conservative estimate of the amount of cations released to solution as a result of microbial activity.

With the exception of Na (Figure 6B), prior microbial DIO oxidation had a small but detectable influence on subsequent organotrophically mediated cation release. In the DIO, Na resides primarily in the mineral plagioclase (Na-Ca feldspar), which is unresponsive to Fe(II) oxidation due to the lack of Fe in the crystal structure. Thus, plagioclase would not be expected to be subjected to crystallographic defects associated with charge imbalance generated by the oxidation of structural Fe (Shelobolina et al., 2012b; Napieralski et al., 2019). A modest increase in the amount of Ca released was observed in DIO-Ox + Gluc-Inoc vs. DIO-Cont + Gluc-Inoc reactors (Figure 5A), although this difference was not outside of the error (range) of duplicate reactors. Plagioclase constitutes ca. 56.4 wt% of the DIO compared to the ca 6.3% of hornblende (White et al., 1998), the two mineral phases in which Ca is concentrated. Thus, the input of Ca from the redox-responsive hornblende is likely to be low compared to that from plagioclase. An enhanced release of Mg was also observed from previously oxidized diorites (Figure 6C). Mg is primarily sourced from the redox active minerals hornblende and biotite, which can account for enhanced release from the oxidized DIO. Biotite is also the major source of K in the DIO. Similar to our previous observations (Napieralski et al., 2019), we observed that K release was repressed when biotite was oxidized (Figure 6D). This surprising effect is best attributed to the assertation by Gilkes et al. (1973a; 1973b) that oxidation of Fe(II) in biotite changes the orientation of biotite hydroxyl groups, creating a more stable environment for the interlayer cation. However, this is not to say that K cannot become depleted from biotite via microbial activity, as K concentrations are clearly higher in DIO-Ox + Gluc-Inoc and DIO-Cont + Gluc-Inoc than in the abiotic, unoxidized control (DIO-Cont + Gluc-Sterile) (Figure 6D). As biotite found in soils is often already at least partially oxidized/altered (Wilson, 2004), this observation is not at odds with the dogma that microbial activity in the rhizosphere plays an important role in soil fertility by increasing K availability. Put more plainly, the biologically enhanced release of K from partially oxidized biotite is greater than the abiotic release of K in fresh, unoxidized biotite, despite the fact that the prior oxidation of biotite repressed the release of K overall in chemolithotrophic experiments. All together this work is consistent with our previous observation that ferromagnesian minerals which have undergone modest surface oxidation are more susceptible acidolysis by mineral acids, and the supposition that FeOB activity may enhance the weatherability of ferromagnesian minerals (Napieralski et al., 2019).

### Potential Metabolic Interactions *in situ*

**FIGURE 8:**
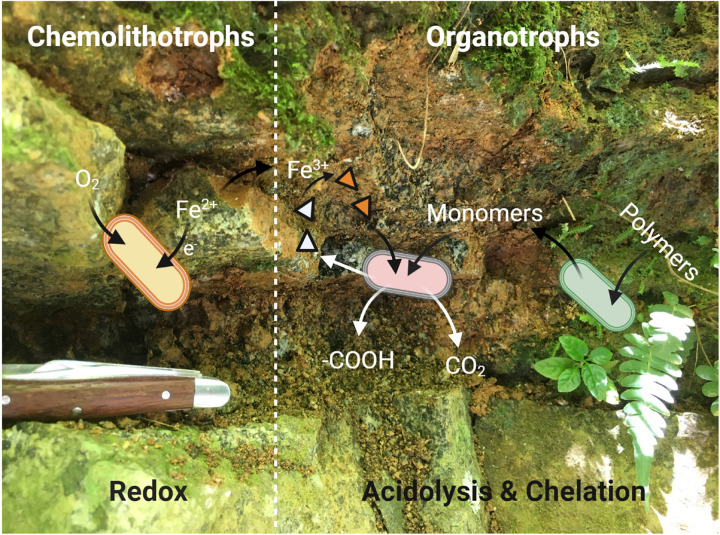
A simplified conceptual cartoon of the potential interactions between members of the “weathering microbiome” of a surficial exposure of the Rio Blanco Quartz Diorite, based on the mechanisms of microbial weathering described by Uroz et al. (2009). Redox based weathering is performed via chemolithotrophic FeOB (yellow cells) which oxidize mineral-bound Fe(II) via EET. The resultant mineralogical transformations render the minerals more susceptible to weathering by organotrophic bacteria (pink and green cells). The organotrophic degradation of complex, plant derived organics provides monomers which can be readily utilized by soil microorganisms, leading to acidolysis by organic and carbonic acids. The Fe(III) generated by FeOB provides a suitable substrate for chelation by siderophores (triangles, empty when unbound) which may be produced by either organotrophic or chemolithotrophic (not shown in diagram for simplicity) taxa.

We have used microbiological and metagenomic analyses to inform the potential metabolic interactions between community members involved in the surficial weathering of the DIO, and a conceptual model is provided in Figure 8. It is clear from the *in situ* metagenomes (GbOC-A and GbOC-B) that degradation of complex organics is an important metabolic pathway. Multiple MAGs, including putative *Mucilaginibacter*, are enriched in carbohydrate active enzymes. *Mucilaginibacter* sp. have been reported to degrade complex organics such as cellulose and hemicellulose and play an important role in degradation of plant biomass in forest soils (López-Mondéjar et al., 2016). While not directly related to weathering, the enzymatic degradation of complex organic carbon to oligosaccharides facilitates the release of plant-derived carbon to the soil solution (Guggenberger et al., 1994). Once depolymerized, plant derived organic carbon would then be available to other regolith organotrophs, including multiple β-proteobacteria also abundant in the *in situ* libraries. β-proteobacteria, particularly the *Burkholderiales*, have been shown to be abundant in weathering systems, and while genomic markers of their ability to enhance mineral weathering are lacking, they have been previously reported to correlate with mineral dissolution *in vitro* (Lepleux et al., 2012; Stranghoener et al., 2018). With the exception of a single *Cupriavidus* MAG, no siderophore biosynthesis pathways were associated with *Burkholderiales* MAGs. This suggests that their ability to enhance mineral dissolution may rely on mechanisms other than chelation by siderophores, such as organic acid production.

Although it might be tempting to think that the availability of carbon would limit chemolithoautotrophic metabolisms, the data presented herein do not necessarily support that assumption. In the early stages of chemolithotrophic incubation, ATP declined across all experimental conditions (Figure 3A), suggesting an overall decline in microbial biomass. However, ATP in DIO free reactors (NoMin and QTZ) remains elevated above the abiotic control for the duration of the experiment, indicating actively metabolizing cells, likely oxidizing residual carbon. Assuming a relatively equal input of carbon occurred during inoculation of each reactor, sufficient carbon would similarly be available in diorite amended reactors. As Fe(II)/Fe(tot) declines over the entire course of the experiment (Figure 3A), and the most abundant MAG obtained from each DIO reactor has the genetic capacity for chemolithoautotrophic growth, it does not seem likely that chemolithoautotrophs were inhibited by the presence of residual carbon and organotrophic activity. This is further supported by slight decline in Fe(II)/Fe(tot) in glucose amended DIO reactors (Figure 5A). It thus seems possible that the putative chemolithoautotrophs, detected in the metagenomic co-assembly can co-exist and metabolize Fe(II) *in situ*, with the oxidative weathering activity of FeOB contributing to the ability of organotrophic microorganisms to further weather ferromagnesian minerals via other mechanisms, including acidolysis and chelation by siderophores. As siderophores are highly specific to Fe(III), with a low affinity for Fe(II), (Neilands, 1995), the implication is that their mechanism of action in enhanced Fe(II) mineral dissolution must almost certainly require prior oxidation, either chemically or biologically, to obtain a suitable substrate. Thus, the oxidation of mineral bound Fe(II) by FeOB may act to increase Fe(III) availability for cellular uptake via siderophores.

## Future Perspectives

We have identified the potential involvement FeOB in silicate mineral weathering in both a subsurface (Napieralski et al., 2019) and surficial quartz diorite weathering environment and have also identified a potential marker for microbial oxidative weathering in Cyc2. In both the present study and our previous work on subsurface weathering, MAGs containing Cyc2 homologs are abundant in Fe(II)-oxidizing enrichment cultures, and the functionality of Cyc2 as an Fe(II) oxidase in neutrophilic FeOB has recently been validated (McAllister et al., 2020). While most studies on *in situ* weathering rely on 16S rRNA gene-based surveys (Liermann et al., 2014; Wild et al., 2019), taxonomic information alone is often not enough to make metabolic inferences, and the role of non-canonical FeOB in terrestrial weathering is likely to be under recognized. Although metagenomic investigations are helping to unravel microbial pathways and biogeochemical implications in a variety of geological habitats (Anantharaman et al., 2016a, b; Fortney et al., 2018; Colman et al., 2019), the terrestrial “weathering microbiome” remains largely unexplored, with the genetic mechanism for biogeochemical weathering only beginning to be revealed (Uroz et al., 2013). This work thus provides a framework and additional genomic target for future investigations into the role of microorganisms in biogeochemical weathering.

## Data Availability Statement

Sequencing data generated in the study, including raw reads for individual metagenomes (BioSamples SAMN15518762-69) have been deposited in the National Center for Biotechnology Information database under the BioProject ID PRJNA645909.

## Author Contributions

SN and ER designed the research. SN conducted the field and laboratory work, performed the data analysis, and wrote the manuscript with input from ER. Both authors contributed to the article and approved the submitted version.

## Conflict of Interest

The authors declare that the research was conducted in the absence of any commercial or financial relationships that could be construed as a potential conflict of interest.
